# The Etiology, Diagnosis, and Management of Cerebrospinal Fluid Rhinorrhea: A Tertiary Center Experience

**DOI:** 10.7759/cureus.42661

**Published:** 2023-07-29

**Authors:** Faisal A Noori, Dalia M Hamdan, Yousef I Alaqsam, Dakheelallah A Almutairi

**Affiliations:** 1 College of Medicine, King Saud Bin Abdulaziz University for Health Sciences, Jeddah, SAU; 2 Medicine, King Abdullah International Medical Research Center, Jeddah, SAU; 3 Otolaryngology, King Abdulaziz Medical City, Ministry of National Guard Health Affairs, Jeddah, SAU

**Keywords:** spontaneous cerebrospinal fluid rhinorrhea, csf rhinorrhoea, traumatic csf leak, cerebrospinal fluid rhinorrhea, endoscopic csf leak repair

## Abstract

Introduction

The aim of the present study was to describe our institution’s nine years of experience in the endoscopic endonasal management of cerebrospinal fluid (CSF) rhinorrhea and to discuss the causes, sites, and outcomes.

Methodology

The medical records of patients diagnosed with CSF rhinorrhea in King Abdulaziz Medical City-Jeddah (KAMC-J) between 2014 and 2023 were retrospectively reviewed, and all relevant information including body mass index, medical and surgical history, and postoperative outcomes were obtained.

Results

A total of 20 cases were included in the present study, sixteen (80%) of which were females and four (20%) were males. The mean age of participants was 42.59±13.9 years. Nine cases (45%) were spontaneous CSF rhinorrhea and 11 (55%) were traumatic; within the traumatic group, six cases (54%) were iatrogenic either following previous neurosurgery or functional endoscopic sinus surgery, while the remaining five cases were related to motor vehicle accidents. The mean body mass index for the spontaneous CSF leak was 32 Kg/m^2^, and 33 Kg/m^2^ for the traumatic leaks, no statistically significant difference was noted. The cribriform plate was the most common site of leakage (65%). A multilayer surgical technique using facia lata graft with nasoseptal flap was the most common choice for reconstruction with a first-attempt success rate approximating 90%. A recurrence was observed in two patients only. No major complications were reported. The average length of stay was nine days.

Conclusion

The endoscopic endonasal repair of CSF leak is a safe and reliable procedure and is associated with high success rates and low risk of complications. Therefore, it should be preferred as a first-line treatment for CSF rhinorrhea.

## Introduction

Cerebrospinal fluid (CSF) is a physiological fluid that forms a protective layer for the brain against acute changes and maintains intracranial pressure [1]. It is secreted by the choroid plexus in the lateral, third, and fourth ventricles and circulates between the arachnoid mater and the pia mater through the subarachnoid space [2]. However, a defect between the subarachnoid space and a pneumatized area in the skull base including the sinonasal tract can lead to CSF leakage. For CSF rhinorrhea to happen, the breach must involve the arachnoid membrane, dura mater, the bony skull base and periosteum, and the nasal mucosa [2]. CSF leakage is usually classified according to the etiology into traumatic and non-traumatic. Traumatic leaks are further subdivided into accidental head injuries and iatrogenic. Penetrating and closed-head trauma are more common and responsible for 80% of all cases of CSF leaks while iatrogenic or post-surgical accounts for only 16%. Spontaneous or non-traumatic CSF leakage accounts for less than 4% of all CSF leaks [3]. Non-traumatic or primary CSF leak occurs in patients without antecedent causes. However, recent evidence suggests that spontaneous CSF rhinorrhea can be a result of elevated intracranial pressure (ICP). Several factors have been associated with increased ICP including obesity, female gender, and obstructive sleep apnea. A study shows that approximately (72%) of patients with spontaneous CSF leaks were female, and approximately 45% had obstructive sleep apnea [4].

The typical clinical presentation of CSF leak is clear, unilateral rhinorrhea, which is sometimes mixed with blood in traumatic etiologies; it is classically exacerbated by bending over or performing a Valsalva maneuver [5]. The presence of headache should alert the clinician to the possibility of elevated intracranial pressure or intracranial pathology [6].

A thorough history-taking remains one of the first and very important steps in the diagnostic algorithm of CSF rhinorrhea as it may uncover a history of trauma, meningitis, or surgery [7]. Confirmation of CSF leak is often needed, and it is done noninvasively through a series of biochemical analyses of CSF markers. CSF glucose is one of the most widely available markers; however, beta-2-transferrin is the most used with a great sensitivity and specificity approximating 97% and 99% respectively [8,9]. As for beta-trace protein, despite carrying the same advantages as beta-2-transferrin, it is not of great clinical utility [10]. The gold standard radiological imaging to definitively localize the site of the leak is typically done through high resolution computed tomography (HRCT) scan. If additional imaging is required, then Magnetic Resonance Imaging (MRI) would also be done as it offers great accuracy in detection of CSF fistulas or sacs [11].

In most cases, traumatic causes of CSF rhinorrhea can be managed conservatively through bed rest with or without placement of a lumbar drain, anti-emetics, stool softeners, and head elevation [12]. However, in some cases, surgery is a necessity to avoid complications like meningitis and brain abscesses [13]. The first surgical repair of CSF leaks was through an intracranial approach as described by Dandy in 1926 [14]; however, craniotomies come with significant morbidity and with recurrence rates as high as 40% [15]. More recently with the advent of endoscopic endonasal approaches in the 1980s, many reports show success rates as high as 95% [16], which has given rise to significant interest in the endoscopic management of CSF rhinorrhea. The goal of endoscopic repair is to achieve complete separation between the pneumatized areas of the sinonasal cavities and the subarachnoid space, thus decreasing the risk of infections and maintaining normal CSF circulation [5]. Multiple different types of graft materials can be used in the repair process, such as fascia lata or nasal-septal grafts.

Very few papers have been published locally, and little is known about the management trends of CSF rhinorrhea in Saudi Arabia [17-19]. In this retrospective cohort, we aim to present the nine-year experience of a major tertiary center elucidating the etiologies, management, and outcomes of CSF rhinorrhea.

## Materials and methods

Study subjects and site

The present study included all patients with clinically and biochemically confirmed CSF rhinorrhea who presented to the department of Otolaryngology-Head & Neck Surgery or Neurosurgery at King Abdulaziz Medical City, Jeddah, Saudi Arabia between 2014 and 2023. The patients’ files were retrospectively reviewed for background data, radiological findings, surgical repair notes, outcome, and postoperative care. Statistical analysis has been performed using Statistical Package for Social Sciences (SPSS) version 29 (IBM Corp., Armonk, NY, USA). The present study has been ethically approved by the Institutional Review Board in King Abdullah International Medical Research Center (KAIMRC) with the number NRJ21J/217/09.

Preoperative care

A thorough clinical evaluation through proper history taking and physical examination was done prior to any investigation. After that, a careful assessment of the nasal cavities was performed in-office utilizing a variety of rigid and flexible nasal scopes. The presence of CSF rhinorrhea was then suspected upon the visualization of clear fluid drainage through the skull base. In some cases, a sample of the fluid was taken and B2 transferrin was then performed to confirm the nature of the fluid. Localization of the leak site was mainly performed by HRCT to visualize the defect site as well as other signs such as empty sella; however, there were some instances where MRI was utilized. All patients in the current study were managed by both Otolaryngology and Neurosurgery teams.

Surgical technique

An endonasal endoscopic repair of CSF leak under general anaesthesia was attempted in all patients in the present study. Firstly, intra-venous antibiotics were given with the induction of anaesthesia, and then nasal mucosa was decongested by lidocaine or epinephrine-soaked tampons. Afterwards, an evaluation of the nasal cavity was done using both 0° and 30° rigid scope. In order to reach the defect site and achieve decent exposure, multiple methods of sinus dissection were employed. For defects in the medial aspects of the cribriform plate, a traditional anterior-posterior ethmoidectomy was performed, while defects in the fovea ethmoidalis or lateral side of cribriform plate require expansion of the ethmoidectomy and partial middle turbinectomy. For defects in the sphenoid sinus, there was no need to disrupt the ethmoidal sinus anatomy. Once the site of the defect is well exposed, preparation of the recipient bed involves careful debridement or cautery of surrounding mucosa and tissue to encourage successful graft integration, repair grafts were prepared and chosen based on the size of the defect. For small defects a two-layer approach was used, while larger defects employed a multilayered repair. A variety of graft materials have been used including fascia lata, pedicled nasoseptal flap, septal cartilage, and middle turbinate mucosa. Preservation of sinus drainage is of paramount importance to prevent postoperative complications such as infection or mucocele. Additionally, any encephaloceles encountered during the process are meticulously reduced and ablated using bipolar cautery, ensuring the safe and effective removal of these abnormal tissue growths. Finally, to achieve impermeable closure fibrin glue (Tisseel) was used between layers.

Postoperative care

After the procedure, all patients were instructed to avoid exertion and were confined to bed rest with head elevation for a minimum of 48 hours. Additionally, measures to prevent increased intracranial pressure were followed such as stool softeners, and instructions to avoid nose blowing, coughing, sneezing with mouth open, etc. Antibiotics were prescribed for all patients who underwent the surgery. The nasal packs were removed after three to five days. On one week follow-up any crustations were removed, and assessment of nasal cavity was reattempted. Frequent follow-ups were then offered for patients to assess for any recurrence through an endonasal endoscope.

## Results

Demographic data

The present chart review identified 20 patients who underwent endonasal endoscopic repair of CSF leak, of which 16 (80%) were females and four (20%) were males. Patients were classified according to etiology of CSF leak into traumatic and spontaneous groups. There were no significant differences in gender among the spontaneous and traumatic groups (p-value=0.369). The mean age of study participants was 42.59±13.9 years. The mean age of the traumatic group was 40.09±15.5 years while the mean age for the spontaneous group was 46.44±11.64 years. There was no statistically significant difference between the two groups regarding age (p-value=0.162). Demographic data and clinical characteristics are summarized in Table 1.

**Table 1:** Demographic and clinical characteristics of patients presenting with cerebrospinal fluid (CSF) rhinorrhea

Serial Number	Age (Years)	Gender	Etiology	Defect Site	Defect Size (mm)	Graft Material
1	45	Male	Traumatic	Sphenoidal	5	Collagen sponge and Tisseel
2	40	Female	Traumatic (Iatrogenic)	Cribriform	5	Fascia Lata with Tisseel fibrin glue
3	26	Male	Traumatic (Iatrogenic)	Cribriform	4	Inferior Turbinate Graft with TachoSil and Nasopore
4	23	Female	Traumatic	Cribriform	6.4	Fascia Lata graft plus Nasospetal flap with Tisseel
5	31	Female	Traumatic (Iatrogenic)	Cribriform	5	Fascia Lata with Nasoseptal Flap
6	22	Female	Spontaneous	Fovea Ethmoidalis	4	Fascia Lata with Tisseel Application
7	74	Female	Traumatic	Cribriform	8	Fascia Lata with Nasoseptal Flap
8	31	Female	Traumatic (Iatrogenic)	Cribriform	5	Fascia Lata with Nasoseptal Flap
9	54	Female	Traumatic (Iatrogenic)	Cribriform	7.6	Fascia Lata with Nasoseptal flap
10	42	Female	Traumatic	Cribriform	3	Fascia Lata with Nasoseptal Flap
11	49	Male	Spontaneous	Fovea Ethmoidalis	4	Tutoplast Fascia Lata supported with Middle Turbinate mucosa.
12	41	Female	Spontaneous	Cribriform	7	Fascia Lata with Nasoseptal Flap
13	24	Female	Traumatic (Iatrogenic)	Cribriform	14	Fascia Lata and nasospetal flap
14	51	Male	Traumatic	Frontal	20	Fascia Lata with Nasoseptal Flap
15	39	Female	Spontaneous	Sphenoidal	5	Collagen sponge and tisseel
16	62	Female	Spontaneous	Cribriform	5	Fascia Lata with Nasoseptal Flap
17	51	Female	Spontaneous	Cribriform plate	4	Fascia Lata with Nasoseptal Flap
18	54	Female	Spontaneous	Cribriform plate	5	Fascia Lata and Tisseel
19	45	Female	Spontaneous	Left sphenoid sinus	7	Fascia Lata with Nasoseptal Flap
20	55	Female	Spontaneous	Roof of the left ethmoid cell	6	Fascia Lata with Nasoseptal Flap

Presentation data

The mean duration of CSF rhinorrhea in all patient groups was 515 days. The mean duration in the traumatic group was 716 days, while in the spontaneous group it was 270 days. There was no significant difference in the duration of CSF leak between the groups (p-value=0.134). A total of 10 (50%) patients had a past medical history of meningitis; four of which were in the spontaneous group and six in the traumatic group. In the traumatic group, four patients had a history of sinus surgery, and three patients had a history of previous neurosurgeries.

Etiology

Traumatic causes of CSF rhinorrhea constituted 55% (n=11) of the entire study population, of which six cases (cases two, three, five, eight, nine, and 13) in Table 1 were iatrogenic following neurosurgical procedures or functional endoscopic sinus surgeries. The remaining five traumatic cases were related to pure trauma such as motor vehicle accidents. On the other hand, there were 45% (n=9) spontaneous cases. No previous repairs had been attempted to any patient in the current cohort.

Size and site of defect

**Figure 1:**
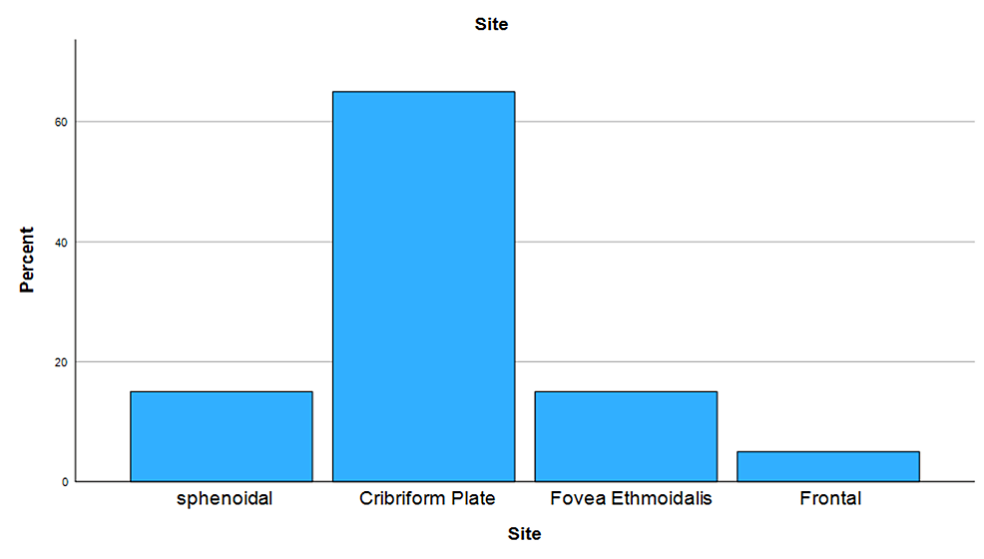
Distribution of cerebrospinal fluid (CSF) leak sites

**Figure 2:**
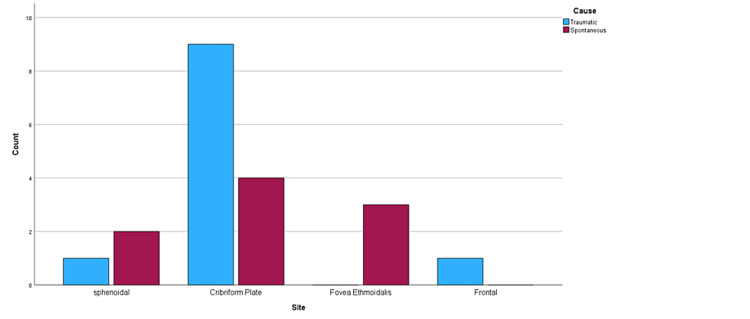
Distribution of cerebrospinal fluid (CSF) leak sites according to etiology of CSF leak

The most common defect site was the cribriform plate (65%; n=13) followed by sphenoidal and fovea ethmoidalis each accounting for 15% (n=3), and the frontal bone was the least (5%; n=1). Eighty-one percent of the anatomical defects in the traumatic group were localized to the cribriform plate, 9.1% in the frontal recess, and 9% in the sphenoidal bone. However, in the spontaneous group, four (44.4%) cases involved the cribriform plate, two cases (22.2%) in the sphenoidal bone, and three cases (33.3%) within the fovea ethmoidalis. There was no statistically significant difference in the distribution of leakage sites between the two groups (p-value=0.106). The mean defect size was 6.5mm±3.9 mm in all patient groups. In the traumatic group the mean defect size was estimated to be 7.5mm±5mm, with a maximum and minimum defect size approaching 20mm and 3mm, respectively. In the spontaneous group, the mean defect size was 5.3mm±1.22mm. There was no significant difference in the defect size between the groups (p-value=0.105). Distribution of defect sites and defect sites according to etiology of CSF leak are shown in Figure 1 and Figure 2 respectively.

Body mass index

**Figure 3:**
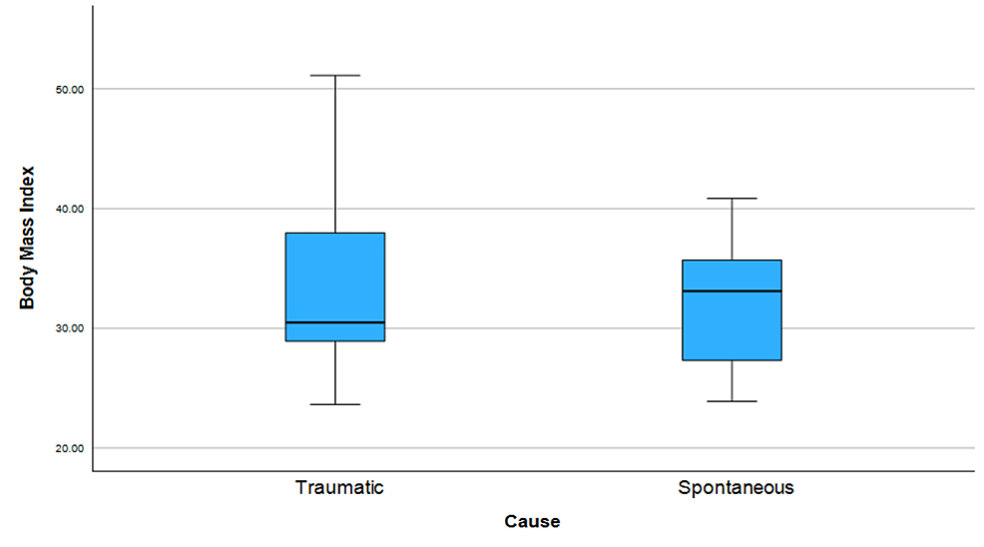
A description of the relationship between body mass index and cerebrospinal fluid (CSF) leak etiology

The mean body mass index (BMI) in the entire population was 33.16±7.2 Kg/m2. In the traumatic group the mean BMI was 33.86±8.6 Kg/m2, while in the spontaneous group it was approximately 32.3±5.3 Kg/m2. There was no significant difference between the two groups (p-value=0.321). The relationship between BMI and etiology of CSF leak is depicted in Figure 3.

Preoperative care

Nasal secretions were sent for B2 transferrin analysis in only nine (45%) cases. The diagnosis was achieved through CT scan in 18 (90%) cases, while MRI was needed only in two (10%) cases (cases 10 and 15) in Table 1.

Surgical management

The overall success rate in our experience is approximately 90% with two cases of recurrent CSF leak recorded. Multiple different graft types were employed for the repair of the defect. In most cases a fascia lata graft with nasoseptal flap was used. In the other cases fascia lata was supported by middle turbinate graft, and in one case an inferior turbinate graft with TachoSil and NasoPore was used for closure of the defect. There were no major postoperative complications recorded except for one case where the patient developed meningitis after the surgery. The hospital stays postoperatively ranged from one day to 56 days with a mean of 9±11 days. The mean follow-up duration postoperatively was two years. There were no significant differences in the follow-up durations between the groups (p-value=0.56).

## Discussion

The present study describes nine years of experience at a tertiary medical center with CSF rhinorrhea. The primary goal of this comprehensive review is to assess all potential causes of CSF rhinorrhea, the diagnostic methods used, the therapeutic strategy for patients at our center, and the prognosis of these patients as well as the recurrence rate. In this review, 20 patients were identified as CSF leak cases and underwent endonasal endoscopic repair. Our study observed that most of them were obese females (80%) which is consistent with the result reported in the literature [20].

In our series, iatrogenic damage accounted for most cases with CSF leak. These findings are in accordance with findings reported by Al-Ghamdi et al. [18]. Post-traumatic CSF leaks induced by surgical or iatrogenic defects are typically induced by a variety of surgical procedures such as endoscopic sinus surgery, otologic surgery, septoplasty, trans-nasal excision of pituitary gland tumors, or other skull base procedures. While prior research suggests that traumatic CSF leaks are more common, Psaltis et al. found that traumatic and nontraumatic etiologies were practically equally prevalent in their study. They also discovered that spontaneous leaks and iatrogenic causes are the most frequent causes of nontraumatic and traumatic CSF leaks, respectively [21].

To prevent complications like meningitis and low-pressure headaches, it is crucial to correctly locate the CSF leak site and source and provide the required treatment [22]. The clinical presentation of the patient greatly guides the proper diagnosis. The symptoms are non-specific, and patients typically present with clear unilateral rhinorrhea, which is exacerbated by Valsalva maneuvers. Before undertaking imaging investigations, some authors recommended in one of the algorithms created for the diagnosis of CSF rhinorrhea that the fluid must be examined for beta-2 transferrin or, when available, beta-trace protein to confirm or rule out the existence of CSF rhinorrhea [23]. In our cohort, beta-2 transferrin analysis was done in nine cases, and it was positive in all of them. However, imaging modalities were the cornerstone for diagnosing and localizing the CSF leak site in our patients. The most common defect site was the cribriform plate (65%). This finding is directly in line with previous findings in the literature [21,24,25].

Surgical treatment of CSF leak can be done by an intracranial or extracranial method. The extent and pathophysiology of the leak guided the decision. Management of CSF rhinorrhea by an intracranial approach has morbidity and failure rates ranging from 20 to 40% [26]. Endoscopic surgery, on the other hand, is less invasive and has a success rate of 90 to 100% [16]. In this cohort, the leak was sealed using an endoscopic method with multiple types of grafts such as fascia lata with nasoseptal flap or middle turbinate graft, and inferior turbinate graft with TachoSil and NasoPore. In our experience, the total success rate is around 90%, with two cases of recurrent CSF leak documented. This result is higher than the result of another report from a hospital in Jeddah, Saudi Arabia (87.5%) [18].

## Conclusions

CSF rhinorrhea is a very rare condition caused by a variety of etiologies, most encountered in middle-aged obese female patients. Due to dangerous complications, the disease must be diagnosed and managed as soon as possible. In our department, iatrogenic trauma was the most prevalent cause of CSF rhinorrhea, and the cribriform plate was the most common defect site. In terms of management, the high overall rate of closure in this study demonstrates that the trans-nasal endoscopic method is a safe and effective surgical therapy for CSF rhinorrhea.
